# Temporal perception deficits in schizophrenia: integration is the problem, not deployment of attentions

**DOI:** 10.1038/srep09745

**Published:** 2015-05-05

**Authors:** Li Su, Brad Wyble, Lai-quan Zhou, Kui Wang, Yu-na Wang, Eric F. C. Cheung, Howard Bowman, Raymond C. K. Chan

**Affiliations:** 1 Department of Psychiatry, University of Cambridge, Cambridge, UK; 2 Neuropsychology and Applied Cognitive Neuroscience Laboratory, Key Laboratory of Mental Health, Institute of Psychology, Chinese Academy of Sciences, Beijing, China; 3 Department of Psychology, The Pennsylvania State University, Pennsylvania, USA; 4 University of Chinese Academy of Sciences, Beijing, China; 5 Castle Peak Hospital, Hong Kong Special Administrative Region, China; 6 School of Computing, University of Kent, Canterbury, UK; 7 School of Psychology, University of Birmingham, Birmingham, UK

## Abstract

Patients with schizophrenia are known to have impairments in sensory processing. In order to understand the specific temporal perception deficits of schizophrenia, we investigated and determined to what extent impairments in temporal integration can be dissociated from attention deployment using Attentional Blink (AB). Our findings showed that there was no evident deficit in the deployment of attention in patients with schizophrenia. However, patients showed an increased temporal integration deficit within a hundred-millisecond timescale. The degree of such integration dysfunction was correlated with the clinical manifestations of schizophrenia. There was no difference between individuals with/without schizotypal personality disorder in temporal integration. Differently from previous studies using the AB, we did not find a significant impairment in deployment of attention in schizophrenia. Instead, we used both theoretical and empirical approaches to show that previous findings (using the suppression ratio to correct for the baseline difference) produced a systematic exaggeration of the attention deficits. Instead, we modulated the perceptual difficulty of the task to bring the baseline levels of target detection between the groups into closer alignment. We found that the integration dysfunction rather than deployment of attention is clinically relevant, and thus should be an additional focus of research in schizophrenia.

Patients with schizophrenia have profound impairments in both the early visual perceptual stage^1^ and the later attentional stage of visual processing^2^. In addition, patients with schizophrenia have been shown to be impaired in informational integration/encapsulation^3^, cross-modality sensory processing^4^,^5^ and temporal binding^6^. Those with schizophrenia also have deficits in auditory tasks that require accurate detection of inter-stimulus interval duration^7^,^8^. Using electrophysiology and steady-state visual evoked potentials, abnormal target identification was found in schizophrenia, however, it seems that the attentional enhancement is intact compared to controls^9^. So, it has been hypothesized that in schizophrenia later impairments in the visual processing stream may account for many abnormalities^10^. In addition, functional MRI studies have found hypo- as well as hyper-connectivity in patients’ brains^11^, and these pathological changes were shown to be associated with psychosis symptoms, suggesting possible neural substrates that may underlie the integration problem in schizophrenia. These consistent findings suggest that schizophrenia patients may have temporal integration deficits during memory encoding, and potentially pathological neuronal changes for distinguishing episodic information. Research on schizophrenia has investigated the transition stage from early perception to the later stage of visual processing focusing on the Attentional Blink (AB) deficit^12^, since the AB can evaluate both the temporal integration of episodic information^13^ and the deployment of attention, two important aspects of temporal processing.

The AB effect refers to a deficit in reporting the second of two targets in rapid serial visual presentation (RSVP) when the second target (T2) is presented between 200–500 ms after the first one (T1) among distractors. There are a number of theoretical accounts for the causes of the AB deficit, such as a second stage bottleneck^14^ and suppression of attention^13^,^15^. However, there is now some agreement that the AB deficit reflects a limitation in the ability to create distinct representations of temporally proximal targets^16^,^17^ and to encode them in the correct order^15^,^18^, and schizophrenia patients may show a great deficit in the AB.

There have been, so far, five published studies on temporal perception deficits in schizophrenia using the AB^19^,^20^,^21^,^22^,^23^. All of them reported a significant impairment of temporal attention in schizophrenia. The implications of these results are that the sensory deficits associated with schizophrenia include impairments in the control of central attentional processes. However, several methodological issues may affect the reliability of these findings. Although most of these studies acknowledged that early visual processing confounds the baseline performance in the AB, which is known to vary with the detectability of targets, none of these studies had matched the baseline level of visual perception of targets. Instead, three of the five studies used a mathematical normalization method to correct for the difference in baseline performance by computing a *suppression ratio*^19^ at the data analysis stage, while the other two did not control for baseline difference. None of them reported temporal integration performance in schizophrenia.

Although the suppression ratio is one of the standard methods used to account for baseline performance difference in the AB, we argue that this highly nonlinear transformation employed in previous studies is not optimal and may result in unreliable conclusions about the temporal processing deficit in schizophrenia. Hence, a more suitable approach is to avoid the confound of visual processing differences, by matching the baseline level of perception in the experiment rather than in the data analysis. We aimed to first demonstrate this through a theoretical assessment of the suppression ratio using a simulation method. We then validated our novel method in two sets of real data on the AB effect: in schizophrenia patients and individuals with schizotypal personality disorder (SPD), which were compared against similarly aged healthy controls. The advantage of recruiting individuals with SPD is that it minimizes the potential impact of medication and illness duration on perceptual and cognitive functioning. When comparing AB performance between patients and controls, and between individuals with SPD and controls (denoted as non-SPDs), we modified a display update parameter in the AB task to match the baseline visual perception scores of the groups. We aimed to determine if there was still a difference in the deployment of attention between these groups when visual perception was matched in this way, and to look for evidence of temporal integration dysfunction in schizophrenia.

## Results

### Theoretical investigation

Schizophrenia patients show significant impairments in early visual processing, which potentially confound the interpretation of the AB effect, i.e. attention deficits. In order to correct for the early perceptual baseline differences, previous studies computed a suppression ratio at each lag, which is expressed in Equation (1).


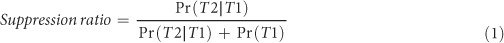


In Equation (1), Pr(*T2*|*T1*) is the conditional probability of correctly reporting the T2 if the participant has also correctly identified T1. Pr(*T1*) denotes the probability of detecting a single target in the RSVP stream. It reflects basic perceptual processing during target detection, and is often significantly impaired in schizophrenia patients^19^. The suppression ratio reflects the magnitude of deviation of Pr(*T2*|*T1*) from Pr(*T1*).

It was claimed that the suppression ratio is a more informative measure of the AB effect, and can remove the overall baseline difference between the two groups revealing the real underlying AB deficit^19^,^22^,^23^. In other words, it was thought to be a direct measurement of the “true” depth of the AB deficit at each lag, i.e. unconfounded by baseline difference. Thus, if there is no difference in the “true” depth of the AB deficit, the suppression ratio should reveal no difference between the two groups. However, AB studies in schizophrenia have all shown that there is still a significant difference in suppression ratios at middle lags, corresponding to T1-T2 target onset asynchronies (TOA) of 200–500 ms, between patients and controls, suggesting that the deficit cannot be simply accounted for by early visual impairments^19^,^22^.

We revisited the above methodology using a simulation-based theoretical analysis based on the method introduced in Cousineau *et al.*^24^. In order to investigate the effect of the suppression ratio, we simulated a situation where the *null hypothesis* holds, i.e. there is no difference in the depth of the AB deficit and only a baseline difference between schizophrenia patients and healthy controls. Hence, we fitted a model (expressed by an inverted Gamma distribution^24^) to the mean performance in the control group’s AB data obtained by Cheung *et al.*^19^, the first published research that used the suppression ratio in schizophrenia. And then, we kept the shape of the curve determined from fitting the model to the controls, and re-fitted it to the mean performance in the schizophrenia group’s data from the same study^19^ by only adding an additional offset to the baseline performance. To model the AB data, we used Equation (2):


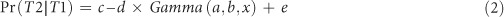


where *Gamma(a,b,x)* denotes the Gamma function with the shape parameter *a* and the scale parameter *b*. Lag is denoted by *x*. The parameters *c* and *d* determine the baseline and the depth of the AB curve respectively. The noise parameter *e* is a random number drawn from a uniform distribution between –0.4 and 0.4, which is set independently for each lag and for each subject. Using nonlinear regression methods^25^, parameters *a*, *b*, *c* and *d* were estimated by fitting the curve to the mean accuracy of the healthy control group from human data in Cheung *et al.*^19^.

In our simulation, the baseline performance Pr(*T1*) was calculated as the average performance of the last two lags. This is because we postulated that T1 accuracy at large TOAs solely reflects basic visual perception, as in the single target RSVP. In all prior AB studies on healthy volunteers and schizophrenia patients, T1 accuracy at large TOAs and single target accuracy have been the same (e.g. Cheung *et al.*^19^).

**Figure 1:**
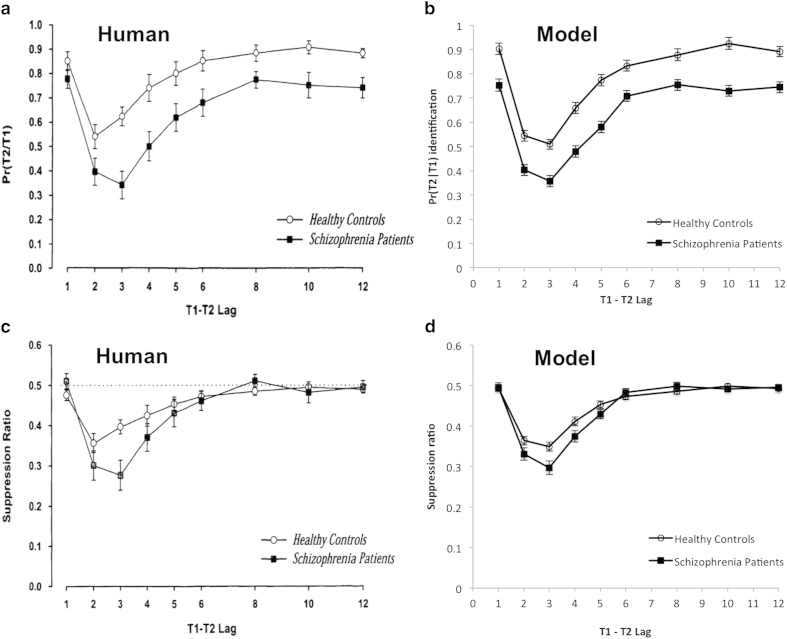
(**a**) Human performance adapted from Cheung *et al.* (19); (**b**) Simulated performance on the AB task; (**c**) Suppression ratio of human data adapted from Cheung *et al.* (19); (**d**) Suppression ratio of the simulated data.

The model fitting was performed using the Matlab Statistics Toolbox (Mathworks). We set the noise parameter *e* to zero while fitting the above equation to the mean performance data of healthy controls^19^. The resulting parameters from the curve fitting for the control group are *a* = 2.1, *b* = 0.96, *c* = 0.86 and *d* = 0.87. We then kept the parameters *a*, *b* and *d* constant in the model and re-fitted the curve to the mean accuracy of the schizophrenia group in Chenug *et al.*^19^ by only varying the baseline parameter *c*. The result of the curve fitting was *c* = 0.7, reflecting a baseline performance decrease of 0.16 from healthy control. Hence, the entire blink curve of the schizophrenia group became lower than that of the control group but the basic shape of the blink curves and most importantly, the mean depth of the blink, are unchanged between groups (see Fig. 1a,b). By randomly re-sampling the noise parameter *e*, we hav*e* simulated the performance of 100 schizophrenia patients and 100 healthy controls to illustrate what would happen in a typical AB experiment. We found that there were significant main effects of group and of lag, but no significant group by lag interaction in our simulated populations. The two groups were significantly different at all lags in a simple effect analysis (paired t-test at each lag) due to the baseline shift in the post hoc analysis.

We then applied the suppression ratio, Equation (1), to the simulated AB data. The simulated results after applying the suppression ratio are shown in Fig. 1d in comparison with the human counterpart from Cheung *et al.*^19^ shown in Fig. 1c. Statistical tests applied on the simulated data revealed that there was a significant main effect of lag, but no main effect of group. Importantly, there was a significant group by lag interaction. In particular, post hoc analysis showed that there was a significant difference in the depth of the blink between the two groups at lag 3 and 4, even though the real difference between the groups was merely a baseline shift in the original data prior to the application of the suppression ratio. Our simulation suggests that the use of the suppression ratio may alter the apparent depth of the blink to create effects that are not present in the original data. In AB experiments, the depth of the blink is an index of attentional deficit. It is based on this theoretical analysis that we question the methodology and results from previous studies that used the suppression ratio. (Indeed, a way to avoid the influence of the baseline difference is to simply and only perform a standard ANOVA, because the group by lag interaction is orthogonal to each main effect, thus, baseline differences are in fact irrelevant to the interaction.)

**Figure 2:**
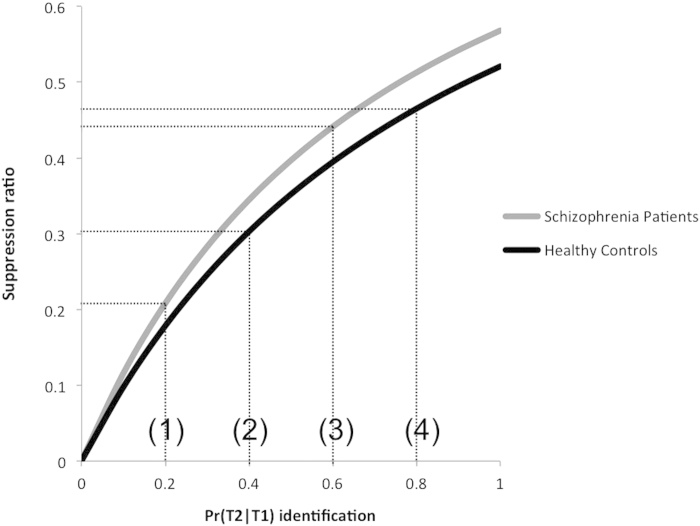
Graphical illustration of the nonlinear suppression ratio.

To fully understand the cause of this problem, we will further analyze the mechanism by which the suppression ratio introduces an interaction that is not in the original data. Fig. 2 shows how the suppression ratio changes as a function of performance and group in a typical AB paradigm. In this illustration, the mean baseline performance Pr(*T1*) is set to 0.76 and 0.92 for the schizophrenia and healthy control groups respectively, according to human data in Chenug *et al.*^19^. It can be seen that if the target identification accuracy Pr(*T2*|*T1*) for the schizophrenia and healthy control groups are 0.2 and 0.4 respectively (c.f. Fig. 2, data points 1 and 2), applying the suppression ratio to the two groups will leave a substantial difference in target detection. This is most likely to happen during the AB, e.g. lag 2, 3 and 4, where target identification accuracy is typically low. In the situation where the task performance is high (e.g. outside the AB), the probability of target detection of both groups will be closer to their baseline performance, i.e. 0.6 and 0.8 (c.f. Fig. 2, data points 3 and 4). In this situation, given the same absolute difference in the original target detection performance as during the AB (i.e. low task performance), the difference in the normalized performance after applying the suppression ratio will now be much smaller. This is due to the nonlinear formula, Equation (1) that is used to compute the suppression ratio, which emphasizes the differences close to floor (i.e. low task performance lags during the AB) and de-emphases the differences close to ceiling (i.e. high task performance lags outside the AB). Hence, such a nonlinear transformation will typically introduce an interaction between lag and group even if it is not present in the actual data.

### Empirical validation 1: schizophrenia patients

**Figure 3:**
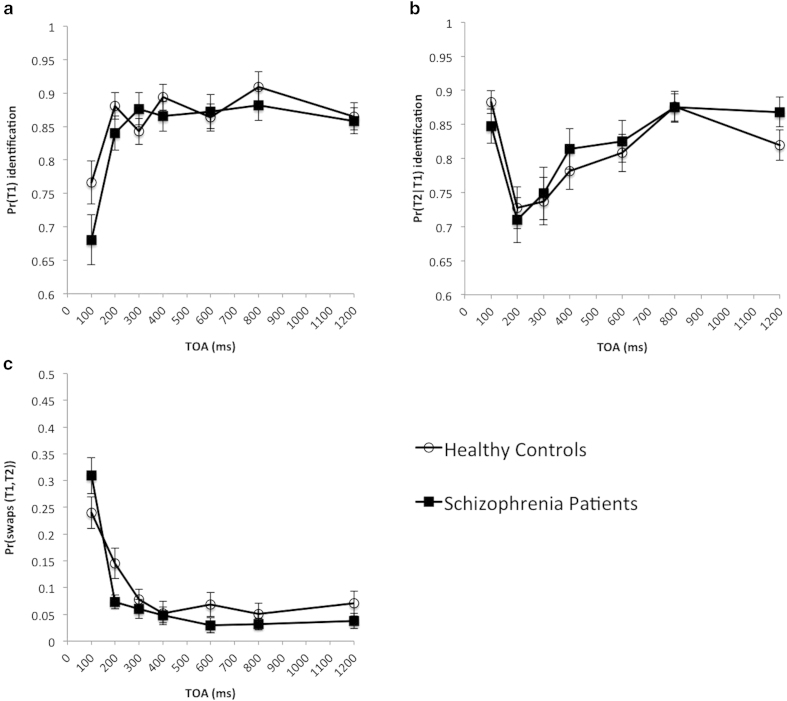
Comparing schizophrenia patients with healthy controls: (**a**) T1 identification accuracy; (**b**) T2 identification accuracy conditioned on correctly reporting T1; (**c**) Temporal order errors for T1 and T2, i.e. swaps.

To test the AB deficit in schizophrenia without using the suppression ratio, we have conducted an experiment, in which patients and controls were matched for age and also matched for baseline performance by modulating the rate of presentation of items within the Rapid Serial Visual Presentation (RSVP) stream. Specially, the stimuli were presented to the control group at twice the speed of the schizophrenia group in order to bring their baseline performance to a close alignment. This is confirmed by an independent t-test, which showed that there was no significant difference between schizophrenia patients and healthy controls for T1 accuracy at 1200 ms TOA, *t*(46) = 0.24, *p* = 0.81. A mixed model ANOVA with lag as a within subject factor and patient status as a between subject factor showed that for T1, there was a significant main effect of lag, *F*(6,276) = 43.86, *p* < 0.0000001, no significant main effect of group, *F*(1,46) = 0.48, *p* = 0.49, and a significant interaction, *F*(6,276) = 4.85, *p* = 0.0001 (see Fig. 3a).

For T2 accuracy contingent on T1, a mixed model ANOVA revealed that there was a significant main effect of lag, *F*(6,276) = 23.2, *p* < 0.0000001, but no significant effect of group, *F*(1,46) = 0.061, *p* = 0.81, and no significant interaction, *F*(6,276) = 1.34, *p* = 0.24 (see Fig. 3b). This is where our finding differs from the significant group × lag interaction reported in previous studies. To further assess whether the non-significant interaction indeed supports the null hypothesis (i.e. that the two groups do not differ in the depth of the blink), we have calculated the Bayes factor^26^ (=0.128) using the ‘BayesFactor’ package in R, version 0.9.7 ( http://cran.r-project.org/web/packages/BayesFactor/) using a mixed model with subject as a random factor and inverse gamma priors. For the fixed effects, the effects for each of the factor levels are assumed to come from a multivariate normal distribution under the constraint that the sum of the effects is 0. The variance of these effects is an unknown multiple of the error variance; the parameter corresponding to this unknown multiple has an inverse two-parameter gamma (0.5,0.25) prior by default. The prior for the random effect levels is similar, but without the sum-to-0 constraint, and to account for the fact that participant variance is robust, the prior on the variance factor is an inverse gamma with larger scale: an inverse gamma (0.5,0.5). Under the null hypothesis that a factor does not account for any variance, all effects for that factor are assumed to be 0. The prior on the error variance σ^2^ under all hypotheses is an improper prior proportional to 1/σ^2^. For details see Rouder *et al.*^26^. This result provides substantial evidence (Bayes factor <1/3) for the null over alternative hypothesis^27^,^28^. In particular, while classical statistics are framed in terms of rejecting the null hypothesis (i.e. if a p-value is below the alpha level), they do not offer an explicit mechanism to affirm the null hypothesis. Bayesian methods, in contrast, do.

We have also analyzed the probability of temporal order errors, as measured by the likelihood of participants correctly reporting both targets, but in the reverse order from their veridical temporal order. Using a mixed model ANOVA, we have found a significant main effect of lag, *F*(6,276) = 59.03, *p* < 0.0000001, no significant main effect of group, *F*(1,46) = 0.53, *p* = 0.47, but a significant interaction, *F*(6,276) = 4.03, *p* = 0.00069 (see Fig. 3c). These results suggest that schizophrenia patients have impairments in temporal integration but no evident AB deficit. (In Appendix A of the Supplementary information, we have shown that the difference in SOAs between groups was unlikely to be an explanation for changes in temporal order errors.)

### Empirical validation 2: schizotypal personality disorder

In order to assess whether this temporal order deficit is also present in subclincal individuals who have some evidence of Schizotypal Personality Disorder, a second experiment was conducted in which individuals with varying scores on the Schizotypal Personality Questionnaire (SPQ)^29^,^30^ were tested with a similar paradigm.

**Figure 4:**
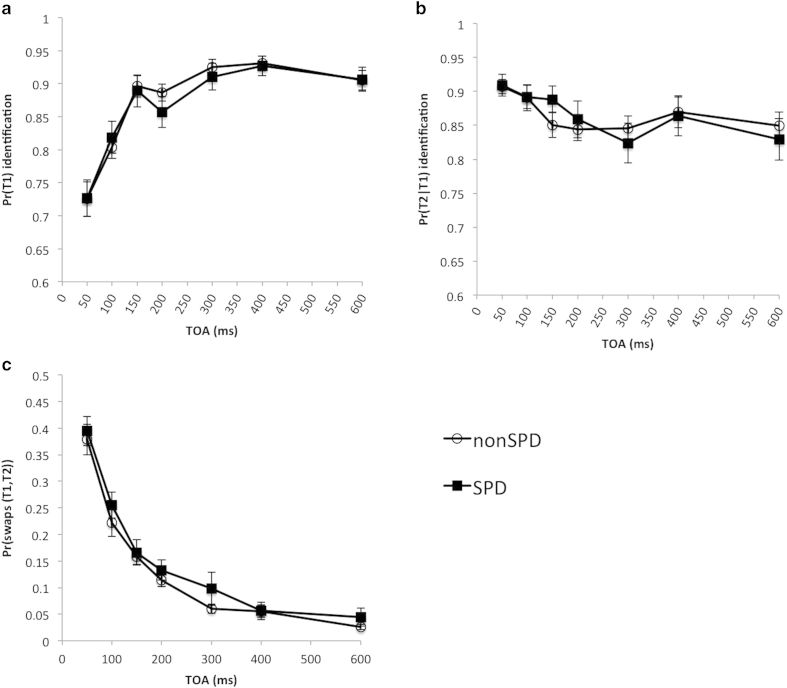
Comparison of SPD with nonSPD: (**a**) T1 identification accuracy; (**b**) T2 identification accuracy conditional on correctly reporting T1; (**c**) Temporal order errors for T1 and T2, i.e. swaps.

As shown in Fig. 4a, there was no significant difference for T1 accuracy at a TOA of 600 ms, *t*(48) = −0.06, *p* = 0.95, indicating that baseline perceptual thresholds were equal between groups. From a mixed model ANOVA of T1 accuracy, we found a significant main effect of lag, *F*(6,288) = 63.49, *p* < 0.0000001, no significant main effect of group, *F*(1,48) = 0.059, *p* = 0.81, and no significant interaction, *F*(6,288) = 0.58, *p* = 0.75. For T2 accuracy contingent on T1, a mixed model ANOVA revealed that there was a significant main effect of lag, *F*(6,288) = 7.84, *p* = 0.0000001, but no significant effect of group, *F*(1,48) = 0.003, *p* = 0.96, and no significant interaction, *F*(6,288) = 1.13, *p* = 0.35, with Bayes factor = 0.132 (see Fig. 4b) providing substantial evidence for the null over alternative hypothesis. For the probability of order errors, a mixed model ANOVA revealed that there was a significant main effect of lag, *F*(6,288) = 91.65, *p* < 0.00000001, no significant main effect of group, *F*(1,48) = 1.32, *p* = 0.26, and no significant interaction, *F*(6,288) = 0.25, *p* = 0.96 (see Fig. 4c).

### Temporal integration deficit in schizophrenia

**Figure 5:**
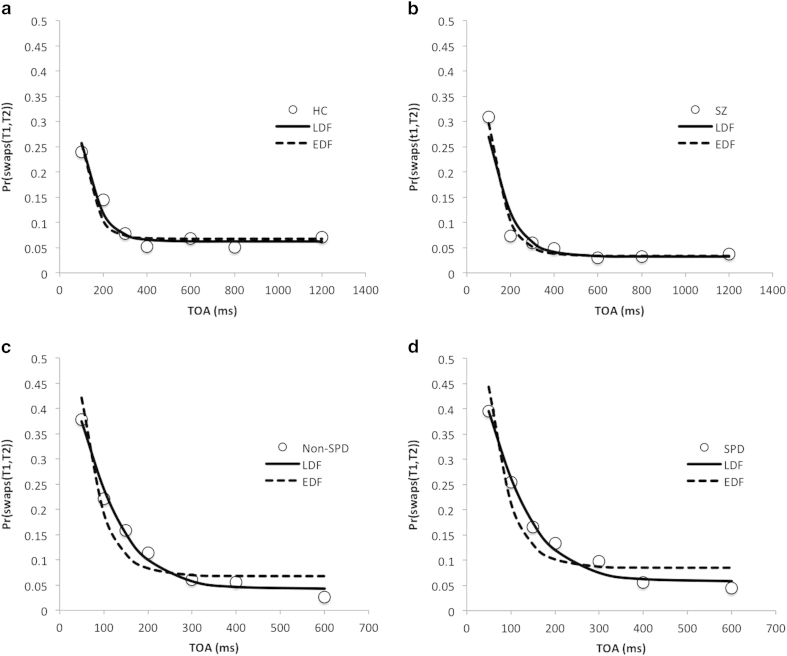
Curve fitting for the swaps. (**a**) healthy controls; (**b**) schizophrenia patients; (**c**) non-SPDs; (**d**) SPDs. The solid curves denote the logistic decay functions and the dashed curves denote the exponential decay functions.

Here, we focused on temporal integration dysfunction in schizophrenia, and performed an analysis of the temporal binding deficits of schizophrenia using all the order error data from both empirical validations. We aimed to demonstrate that the schizophrenia group has a qualitatively different temporal order error pattern to all other groups. This was done by fitting a logistic decay function (LDF) and an exponential decay function (EDF) to the temporal integration deficit data, which can be seen to follow a decay function as the lag increases. The logistic decay function (LDF) is expressed in Equation (3):


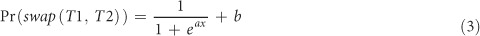


where *Pr* denotes the probability of a subject reporting the two targets in the wrong order, reflecting temporal perception deficits. The parameter *a* sets the decay rate (i.e. the speed as lag increases at which the temporal binding errors reduce to the baseline) and *b* sets the baseline. *x* denotes the lag. The exponential decay function (EDF) is expressed in Equation (4):


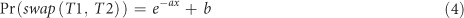


where *a* and *b* are the decay rate and baseline parameters respectively as in the LDF. The LDF and EDF are often compared when modeling population growth/decay^31^. It can be seen that the two models have the same number of parameters, and hence the same complexity, with standard measures such as Bayesian Information Criterion. So, no correction for model flexibility is incorporated in comparing model fits. We fitted both functions to all the groups, and focused on selecting which best explains the data by minimizing a goodness of fit measurement MSE, i.e. mean squared error. We are interested in whether there is a qualitative rather than quantitative difference between groups, i.e. groups are different in terms of which function describes its behavior best. Here, a quantitative difference refers to differences between groups in terms of their parameters for the same function. The results of the curve fitting are shown in Fig. 5.

We found that the LDF provided a better fit than the EDF for non-SPDs, SPDs and healthy controls, but not for patients with schizophrenia. In contrast, the EDF fitted better for schizophrenia patients, but not for other groups, see Table 1. Thus, it seems that schizophrenics exhibit a qualitatively different order error pattern than all other groups, whether those groups have equal perceptual baseline acuity (healthy controls) or equalized SOA (both SPDs and non-SPDs).

**Figure 6:**
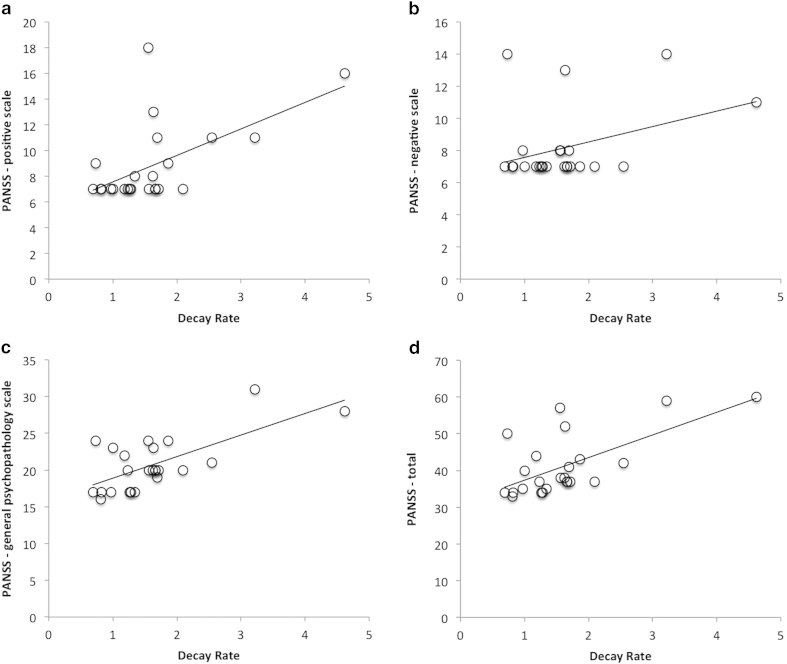
Correlation between the decay rate for each patient and the clinical manifestations of schizophrenia indicated by PANSS scores - (**a**) positive, (**b**) negative, (**c**) general psychopathology and (**d**) total.

We have also fitted the temporal binding errors for each individual patient to the EDF and correlated the resulting decay rates with patients’ clinical symptoms. We found a significant linear correlation between the decay rate parameter and the positive subscale score of PANSS, *r*(22) = 0.58, *p* = 0.0029, the general psychopathology subscale score of PANSS, *r*(22) = 0.68, *p* = 0.002, and the total PANSS score, *r*(22) = 0.64, *p* = 0.0008, but not for the negative subscale of PANSS and other variables including age, IQ, education, logic/visual memory, CPZ equivalent, illness duration and performance on the N-back task. See Fig. 6 for correlation with the PANSS.

## Discussions

The results from this study are different from previous findings on temporal attention deficits in schizophrenia, showing no significant impairment in the AB in the dual-target task. Most importantly, we found that the depth of the blink curve was unaffected in patients with schizophrenia compared with controls when early visual perception was controlled for. This finding is theoretically supported by a critical assessment of the standard suppression ratio method. Our simulation has shown that the suppression ratio may introduce interactions between lag and group that are not present in the original data. Our results put the established view that patients with schizophrenia have both impaired short-term visual memory and deficits in the temporal allocation of attention in question^32^,^33^. In addition, we have found that the AB, and temporal binding errors were uncorrelated with education, IQ and the N-back memory task performance when the early perceptual processing was controlled for. We also found no significant correlation between the clinical manifestations and age, education, IQ, CPZ equivalent doses and illness duration.

Although we did not find a specific AB deficit in patients with schizophrenia, our analysis of temporal order errors revealed a significant deficit specifically in temporal integration rather than late attentional impairment in schizophrenia patients. This integration deficit manifested as a sharper increase in temporal binding errors in schizophrenia patients when the TOA was decreased. Although this temporal integration dysfunction has been overlooked by all previous research on AB in schizophrenia, we suggest that the significant impairment in processing temporal order information at the one hundred-millisecond time scale may be related to several cognitive deficits of schizophrenia. For example, schizophrenia patients have been found to have poorer time perception, temporal coding and binding^6^,^34^,^35^. Temporal order impairments were found to be correlated with the patients’ PANSS positive and general psychopathology ratings, thus may also be important in the clinical manifestations of schizophrenia.

It is also worth considering how these findings can be explained in the Simultaneous Type/Serial Token (STST) modeling framework^13^,^15^, which models a large spectrum of AB and related data. In particular, a specific temporal order deficit suggests some impairment in the capacity to ascribe episodic contexts to experiences. In STST, such contexts are realised with neural structures called tokens. If targets are sufficiently spaced in time, the STST token mechanism correctly allocates different stimuli to different tokens/contexts. In this way, it is able to regenerate the order in which the stimuli occurred, when retrieving from working memory. The resolution of this episodic binding system is determined by the time it takes to associate a token with the representation of a stimulus. One can view the probability of making order errors at short TOAs (particularly lag-1) during dual target RSVP, as an index of this resolution. Thus, an increase in such errors, suggests a loss of accuracy in associating stimuli/experiences with episodic contexts or a loss of episodic distinctiveness^13^. This may also account for the increased rate of temporal binding errors we observed at lag-1 for schizophrenia patients.

In order to generalize our theory of schizophrenia to less serious subclinical samples, we have conducted experiments with individuals with or without SPD. The results showed that there is no difference between SPDs and non-SPDs in terms of early visual processing, attentional processing in AB and temporal binding. We only detected an inconclusive trend for more temporal binding errors for SPD although those SPD subjects were only psychometrically defined using SPQ, and future studies may benefit from recruiting clinically defined SPD subjects. The current study also suffers from the limitation of using behavioral measures only. More direct physiological measures, such as EEG or neuroimaging techniques, may provide better indications of the cognitive deficit in SPD, e.g. Modinos *et al.*^36^.

## Methods

### Participants

In Empirical validation 1, twenty-four in-patients, who are assessed by qualified psychiatrists, meeting DSM-IV^37^ diagnostic criteria for schizophrenia were recruited from the Mental Health Center of Shantou University. A total of 24 healthy individuals matched by age were recruited from the community through advertisements. Clinical symptoms were rated using the Positive and Negative Syndrome Scale (PANSS)^38^. The exclusion criteria for all participants included a past history of head injury, neurological diseases, alcohol/substance dependence, and other concomitant DSM-IV axis I or axis II disorders. Intellectual function was estimated using the short form (Information, Arithmetic, Similarity, and Digit Span) of the Chinese version of the Wechsler Adult Intelligence Scale-Revised (WAIS-R)^39^. Working memory was assessed by the N-back test^40^. Verbal memory and visual memory were assessed by the logical memory and visual reproduction subscales of the Wechsler Memory Scale Revised Chinese version^41^. Demographic information and neuropsychological characteristics of these participants are summarized in Table 2.

In Empirical validation 2, seven hundred university students were administered the SPQ. According to the SPQ manual^29^, people scoring in the top 10% of the SPQ distribution (i.e. 36 points) can be considered as exhibiting schizotypal personality disorder (SPD) traits. As a result, 25 students were identified as having SPD traits and 25 of those scoring around the average SPQ score (i.e. 21 points) were randomly selected to form the control group (non-SPD). The two groups did not differ significantly in gender ratio, age, and IQ (see Table 3).

All participants were right-handed, and none of them had a personal or family history of psychosis, depression, suicide, epilepsy or drug abuse. All experiments were performed in accordance with relevant guidelines and regulations and were approved by the ethics committees of the Institute of Psychology, Chinese Academy of Sciences and the Mental Health Centre of Shantou University. Written informed consent was obtained from all the participants before the administration of the tests.

### Procedures

The experiment was run on a PC using Matlab and the Psychophysics Toolbox-3 extension^42^. Alphanumeric characters were presented in black on a grey background at a distance of 70 cm on a 14.1-inch LCD screen with a 1280 × 800 resolution at a 60 Hz refresh rate.

In Empirical validation 1, a version of the AB task was specifically designed for Chinese language speakers with targets drawn from eight digits with 16 capital letters serving as distractors. In three-quarters of the trials, there were two targets and T1 was followed by T2 at TOAs of 100, 200, 300, 400, 600, 800 and 1200 ms in the RSVP stream. In one-quarter of the trials, there were three targets and the last target was always presented 800 ms after T2. These triple-target trials were excluded from the subsequent analysis. They were designed to ensure that the participants were attending to the stimuli throughout the dual target part of the stream, even in the rare occasion in which T2 was 1200 ms after T1.

The stimulus onset asynchrony (SOA) between two adjacent items in the RSVP stream was set at 100 ms for schizophrenia patients, and 50 ms for healthy controls. This was determined by a pilot study that matched the baseline performance outside of the AB time window, i.e. T1 accuracy at an 1200 ms TOA, between the two groups by presenting stimuli at a different rate, while maintaining the TOA between T1 and T2. In the pilot study, we first explored the possible SOAs and found that for schizophrenia patients an SOA of 100 ms gave a mean T1 accuracy between 0.8 and 0.9 at an 1200 ms TOA. Then we reduced the SOA from 100 ms in a fixed step of 10 ms for controls in order to obtain T1 accuracy at a comparable range to the patients. We found that SOA of 50 ms or, in other words, halved the presentation rate of the schizophrenia patients can compensate for the early visual perceptual differences so that we could avoid the use of the suppression ratio in our data analysis. Participants in both groups were instructed to fixate at the centre of the screen at the beginning of each trial, and report all targets at the end of the stream in the order they were perceived, by typing the digits on the keyboard.

In Empirical validation 2, we used the same design as Empirical validation 1, but based on our pilot study, stimuli were presented to both the SPD and the non-SPD groups at the same SOA of 50 ms. This presentation rate gave the same baseline performance between the two groups. In Empirical validation 2, T2 was presented at TOAs of 50, 100, 150, 200, 300, 400, and 600 ms in the RSVP stream.

## Author Contributions

LS designed the experiment, analyzed the data and wrote the manuscript; BW co-designed the experiment and commented on the manuscript; LQZ, KW and YNW collected the demographic, clinical and behavioral data and processed the data; EFCC commented on the manuscript; HB and RCKC co-designed the experiment and commented on the manuscript. LS and RCKC obtained the financial support and oversaw the study.

## Additional Information

**How to cite this article**: Su, L. *et al.* Temporal Perception Deficits in Schizophrenia: Integration is the problem, not Deployment of Attention. *Sci. Rep.* **5**, 09745; doi: 10.1038/srep09745 (2015).

## Supplementary Material

Supplementary Information

## Figures and Tables

**Table 1:** Curve fitting for the temporal binding errors.

**Group**	**Decay function**	**Decay rate**	**Baseline**	**MSE**
Patient	LDF	1.2	0.03	0.00078
	EDF	1.3	0.03	0.00025
Healthy control	LDF	1.4	0.06	0.00030
	EDF	1.7	0.07	0.00052
SPD	LDF	1.4	0.06	0.00021
	EDF	2.0	0.08	0.00178
Non-SPD	LDF	1.4	0.04	0.00020
	EDF	2.1	0.06	0.00155

LDF: logistic decay function, EDF: exponential decay function, MSE: mean squared error.

**Table 2:** Demographic and neuropsychological characteristics of schizophrenia patients and their control group.

	**Schizophrenia (N = 24)**	**Healthy controls (N = 24)**	**t**	**P**
Age	27.04 (7.35)	28.08 (6.51)	0.52	0.61
Gender (F/M)	8/16	9/15		
Education	10.25 (3.18)	10.79 (2.86)	0.62	0.54
IQ estimates	90.29 (21.86)	104.50 (10.55)	2.87	0.006
Logic memory (in time)	7.29 (4.95)	13.04 (4.55)	4.19	<0.001
Logic memory (delayed)	5.50 (4.66)	10.96 (5.23)	3.82	<0.001
Visual memory (in time)	18.79 (3.64)	20.58 (3.32)	1.78	0.08
Visual memory (delayed)	18.13 (4.01)	20.29 (3.57)	1.97	0.054
0-back ACC	0.88 (0.19)	0.95 (0.10)	1.61	0.11
0-back RT	621.51 (130.80)	537.19 (141.22)	−2.05	0.047
1-back ACC	0.32 (0.23)	0.53 (0.29)	2.59	0.01
1-back RT	765.71 (280.60)	751.18 (246.55)	−0.17	0.87
2-back ACC	0.19 (0.14)	0.36 (0.22)	2.98	0.005
2-back RT	652.33 (206.96)	628.00 (205.74)	−0.38	0.71
PANSS_P	8.83 (3.06)			
PANSS_N	8.17 (2.30)			
PANSS_G	20.71 (3.70)			
PANSS_S	3.25 (0.90)			
PANSS_T	41.17 (8.31)			
Duration	6.74 (5.53)			
CPZ Equi. Doses mg/day	457.29 (251.68)			

**Table 3:** Demographic characteristics of SPD and non-SPD groups.

	**SPD (N = 25)**	**Non-SPD (N = 25)**	***t***	***P***
Age	20.08 (1.04)	20.04 (1.31)	−0.12	0.91
Gender (F/M)	8/17	10/15		
Education	13.76 (0.97)	12.84 (0.85)	−3.57	0.001
IQ estimates	126.61 (10.65)	126.71 (9.36)	0.03	0.97
SPQ_Cognitive perception	17.88 (5.39)	4.64 (3.13)	−10.62	<0.001
SPQ_Interpersonal	12.44 (4.18)	2.60 (1.94)	−10.67	<0.001
SPQ_Disorganization	9.36 (2.14)	1.84 (2.04)	−12.74	<0.001
SPQ_Total score	40.84 (6.61)	9.16 (5.06)	−19.03	<0.001
