# Supplementary material for: Temporal perception deficits in schizophrenia: integration is the problem, not deployment of attentions
Source: Sci Rep. 2015 May 5;5:9745. doi: 10.1038/srep09745 (PMC4419531; doi:10.1038/srep09745)
Supplement: Supplementary Information [file srep09745-s1.doc]

# *Supplementary information*

# **Temporal Perception Deficits in Schizophrenia: Integration is the problem, not Deployment of Attention**

Li Su, Brad Wyble, Lai-quan Zhou, Kui Wang, Yu-na Wang, Eric F. C. Cheung, Howard Bowman, Raymond C. K. Chan

**Appendix A Influence of group SOA differences on temporal order errors**

We found that patients with schizophrenia have a temporal binding deficit reflected by a significant group by lag interaction. However, this difference in order errors may be confounded by the different SOAs used for the two groups; that is, the healthy controls but not the patients saw an intervening distractor between T1 and T2 at lag-1. Unfortunately, this confound cannot be avoided in the current study because there is a conflict between the need to keep the same SOA for both groups in order to make an unbiased comparison for the order errors, and our main objective of controlling for differences in early perceptual processing by reducing the SOA for the control participants.

However, we believe that the intervening distractor would in fact increase the frequency of temporal order errors rather than reduce them. This is supported by a prominent theory of temporal attention: the Simultaneous Type / Serial Token model (STST)[[1]](#endnote-2),[[2]](#endnote-3), which models a large spectrum of AB and related data. This prediction was confirmed by additional data collected for healthy controls. Here, the same SOA of 100ms as the patients was used in the task.

The eSTST model, which is an elaboration of the earlier STST model, predicts that increasing the speed of the RSVP stream to 50ms per stimulus will actually increase, rather than decrease the number of temporal order errors at a 100ms TOA. This prediction stems from the fact that the decreased duration of the targets, coupled with the ~100ms time course of attentional deployment in the model, favors processing of the T2 when the duration of the targets is decreased below 100ms. As a result, the reduction of the SOA used to balance the baseline accuracy between the healthy controls and schizophrenia participants is predicted to increase the number of temporal order errors at a TOA of 100ms, all else being equal.

To test the predictions from the eSTST model, we have collected an additional dataset from 10 healthy participants using the same 100 ms SOA as the patient group. Although we were not able to match their baseline performance as we did in Study 2, the measurement of the temporal order errors was not confounded, allowing an unbiased comparison for the temporal order errors. The 10 healthy controls participated in the experiment in Study 2 first and then were given a break for 10 minutes before being tested again using a longer SOA.

The performance of the additional test is shown in Figure S1A. It can be seen that when there is no intervening distractor between T1 and T2, the probability of order errors was indeed decreased. In comparison to this new control data, patients with schizophrenia showed a clearer increase in order errors at lag-1, see Figure S1B.


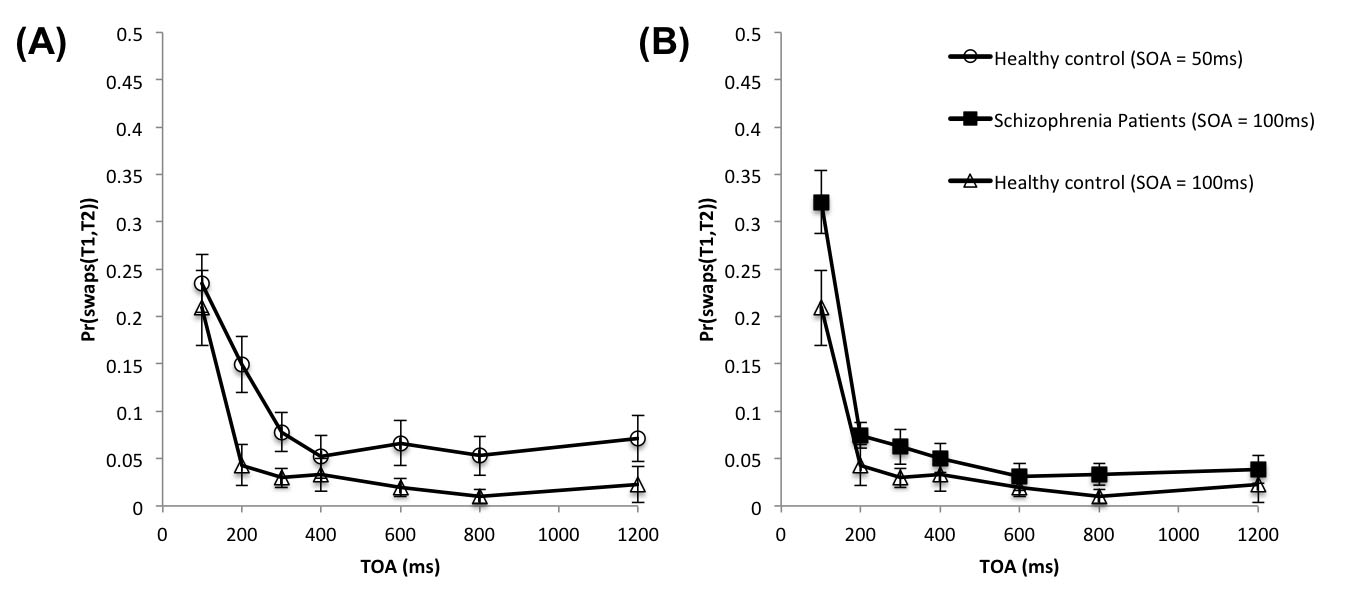


Figure S1 Temporal order errors for T1 and T2 swaps. (A) Healthy controls using 50 ms and 100 ms SOA in the AB task; (B) Comparison between healthy controls and patients with schizophrenia using the same SOA of 100ms.

**Reference**

1. . Bowman, H. & Wyble, B. The Simultaneous Type, Serial Token Model of Temporal Attention and Working Memory. *Psycho. Review* **114**: 38-70 (2007). [↑](#endnote-ref-2)
2. . Wyble, B., Bowman, H. & Nieuwenstein, M. The Attentional Blink provides Episodic Distinctiveness: Sparing at a Cost. *JEP: HPP.* **35**: 787-807 (2009). [↑](#endnote-ref-3)
